# The Oxygenation Index compared with the P/F ratio in ALI/ARDS

**DOI:** 10.1186/cc10698

**Published:** 2012-03-20

**Authors:** M Van Haperen, PH Van der Voort, RJ Bosman

**Affiliations:** 1 AMC, Amsterdam, the Netherlands; 2 Olvg, Amsterdam, the Netherlands

## Introduction

The usual way to describe the severity of pulmonary dysfunction in ventilated ICU patients is by using the PaO_2_/FiO_2 _ratio (PF). The PF may be adjusted by the ventilator pressure settings in order to reduce inspiratory oxygen fraction but the PF does not take the mean airway pressure (MAP) into account. In contrast, the Oxygenation Index (OI) is defined as the reciprocal of PF times MAP: OI = (FiO_2_×mean airway pressure)/PaO_2_. As such, the OI is a better representative of oxygenation dysfunction. The objective was to study the correlation between and the impact of the MAP on the PF and OI.

## Methods

We performed a retrospective analysis of 27 consecutive mechanically ventilated patients admitted to our ICU with bilateral interstitial/alveolar lung disease, defined as ALI or ARDS. The data of these patients were collected during a time period of maximum 30 consecutive days. Demographic data were recorded and the PF, OI and MAP were assessed daily at 6:00 am during the first 30 days of admission. OI >8.1 is usually regarded as ARDS and >5.3 as ALI [1].

## Results

**Figure 1:**
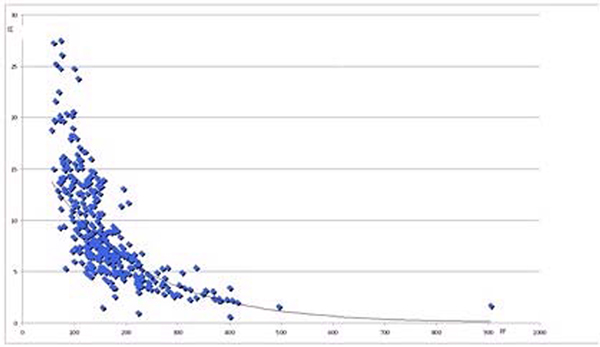
**Oxygenation Index versus P/F ratio**.

We included 27 patients, 25 were male, the mean APACHE II score was 22, the median length of stay on the ICU 11 days and the ICU mortality was 11/16 (69%). The mean PF was 165 (SD 83), the mean OI was 8.2 (SD 5) and the mean MAP was 16 cmH_2_O (SD 5). The 27 patients resulted in 364 measurements. Of these measurements 158 had OI >8.1, of which 157 had PF <200 and a mean MAP of 19.3 cmH_2_O. In one patient PF was >200 while OI was >8.1 with MAP 18 cmH_2_O. Of the 100 measurements with OI 5.3 to 8.1, 14 had PF 200 to 300 and 85 had PF <200. The MAP in these measurements was 17, 64 and 24 cmH_2_O respectively. Figure 1 shows the nonlinear relation between OI and PF.

## Conclusion

In patients with ARDS, OI >8.1 is usually in agreement with PF <200. However, patients with ALI based on OI 5.3 to 8.1 frequently had PF <200. More studies are needed to determine the optimal level of OI for the diagnosis of ALI/ARDS.
